# Evaluation of 3D Modeling Workflows Using Dental CBCT Data for Periodontal Regenerative Treatment

**DOI:** 10.3390/jpm12091355

**Published:** 2022-08-23

**Authors:** Styliani Verykokou, Charalabos Ioannidis, Christos Angelopoulos

**Affiliations:** 1 Laboratory of Photogrammetry, School of Rural, Surveying and Geoinformatics Engineering, National Technical University of Athens, 15780 Athens, Greece; 2 Department of Oral Diagnosis and Radiology, School of Dentistry, National and Kapodistrian University of Athens, 11527 Athens, Greece

**Keywords:** CBCT, cone beam computed tomography, 3D modeling, segmentation, periodontal treatment, scaffold, periodontitis, dental applications

## Abstract

The cone beam computed tomography (CBCT) technology is nowadays widely used in the field of dentistry and its use in the treatment of periodontal diseases has already been tackled in the international literature. At the same time, advanced segmentation methods have been introduced in state-of-the-art medical imaging software and well-established automated techniques for 3D mesh cleaning are available in 3D model editing software. However, except for the application of simple thresholding approaches for the purposes of 3D modeling of the oral cavity using CBCT data for dental applications, which does not yield accurate results, the research that has been conducted using more specialized semi-automated thresholding in dental CBCT images using existing software packages is limited. This article aims to fill the gap in the state-of-the-art research concerning the usage of CBCT data for 3D modeling of the hard tissues of the oral cavity of patients with periodontitis using existing software tools, for the needs of designing and printing 3D scaffolds for periodontal regeneration. In this context, segmentation and 3D modeling workflows using dental CBCT data that belong to a patient with periodontitis are evaluated, comparisons between the 3D models of the teeth and the alveolar bone generated through the experiments that yielded the most satisfactory results are made, and an optimal and efficient methodology for creating 3D models of teeth and alveolar bone, especially for being used as the basis for generating bioabsorbable 3D printed scaffolds of personalized treatment against periodontitis, is discussed.

## 1. Introduction

A few decades after the development of the first commercially available computed tomography (CT) scanner in the early 1970s, the first cone beam computed tomography (CBCT) scanner was developed in 1998 by Mozzo et al. [1] specifically for dental use, representing one of the greatest revolutions in medical imaging. Since then, the CBCT technology was gradually introduced in radiology and became widely used in the fields of dentistry, such as for implant design, periodontal defects, endodontics, and orthodontics, as well as in maxillofacial applications [2,3].

The 3D morphology of a periodontal defect is difficult to be accurately determined through 2D digital X-rays [4]. Thus, the use of CBCT in the diagnosis of periodontal diseases has been already proposed in the international literature [5,6]. Studies have shown that CBCT data are more suitable than X-rays in detecting certain periodontal defects [7,8,9,10,11,12,13,14]. However, the aforementioned studies were performed using 2D CBCT images. Although CBCT images provide cross-sections in multiple orientations (sagittal plane, coronal plane, axial plane), the true morphology of the periodontal defect is not directly visible through them. Therefore, the creation of 3D models for parts of the oral cavity for both periodontal diagnosis and for the design of the appropriate treatment has already been proposed [15,16]. However, in the above studies, a simple thresholding segmentation method was exclusively used, resulting in the production of 3D models of high noise and low accuracy. The deterioration in accuracy and the existence of noise in the 3D models that result from automated segmentation procedures (such as simple thresholding) may be due to several factors, such as artifacts, and tooth characteristics that differ from normal anatomy (e.g., periodontal bone defect).

In addition to simple thresholding, other automated methods have been used for the segmentation of CBCT data for dental applications, such as the use of morphological operators in combination with the Watershed algorithm [17], the level set method [18], and convolutional neural networks [19]. In particular, the methodology proposed by Sepehrian et al. [17] for teeth segmentation in individual “optimal” CBCT images, i.e., images with the greatest number of pixels that belong to teeth, is summarized as follows. A maximum intensity projection (MIP) is used for considering only teeth and bony areas from the initial CBCT slice; the morphological operator’s image filling and closing are sequentially applied to the CBCT slice that is cropped using the MIP mask, so that it is enhanced; finally, the Watershed algorithm is applied to separate each tooth, while using anatomical constraints, to overcome the problem of over-segmentation. However, the aforementioned segmentation methodology is applied to individual CBCT images and not to all of them. In order to extract 3D information from the CBCT data using such a technique, it is required to combine it with additional methods for segmentation of the entire CBCT dataset, which needs further investigation.

The research conducted by Gan et al. [18] focused on the segmentation of teeth and alveolar bone from CBCT images for their 3D reconstruction. According to their study, the segmentation of teeth and alveolar bone in CBCT images remains a challenge, due to their similar intensities, the close position of adjacent teeth, and the complex topological structure of both teeth and alveolar bone. The method includes two main steps: (a) extraction of the connected area of the teeth and the alveolar bone from CBCT images using a level set method; and (b) separation of the individual teeth and the alveolar bone based on the Radon transformation [20] and a local level set method. The experimental results showed that the proposed method yielded successful results in the segmentation of teeth and alveolar bone from high-precision CBCT images, being suitable for the scope of their 3D modeling.

Except for the methodology presented by Gan et al. [18], additional research has been conducted for the use of a level set method for the segmentation of teeth from CBCT data. The works presented by Gao and Chae [21] and Ji et al. [22] constitute typical examples. A level set method for segmentation of teeth on CT data was implemented by Hosntalab et al. [23] and a level set method to segment tooth roots using CT data was applied by Yau et al. [24].

Deep machine learning techniques were applied by Cui et al. [19] for teeth segmentation using CBCT images for 3D reconstruction of the teeth. The methodology followed for each CBCT slice is based on the use of a two-stage neural network as follows: (a) edge detection on CBCT data using neural networks to enhance contrast across borders, and (b) image segmentation for teeth extraction based on the generated neural network. Research on the use of neural networks for the segmentation of teeth and bony areas in CBCT images containing a high percentage of artifacts due to metal objects was conducted by Minnema et al. [25].

As far as the usage of existing segmentation software is concerned, except for the application of a simple thresholding approach for the generation of a 3D model of the oral system using CBCT data for dental applications, the research that has been conducted using more specialized semi-automated segmentation methods in dental CBCT is limited. The study conducted by Palkovics et al. [4] used both semi-automatic and manual tools provided by the open-source medical image editing software 3D Slicer [26] for segmentation of alveolar bone and teeth using CBCT data. The “level tracing” tool (which is used to define an area where the pixels have the same intensity value as a selected pixel) was applied to manually define the alveolar bone in every fourth slice. The “fill between slices” tool was then applied to automatically compute the area of the alveolar bone into slices in which it was not manually defined. This tool uses morphological interpolation [27] for automatic segmentation, based on a user-defined segmentation in some slices. Further improvement of the individual sections was accomplished via manual segmentation tools provided by the software. The same procedure was repeated for the teeth. Three-dimensional polygonal models were created based on the alveolar bone and teeth segmentation and were exported in STL format. Further optimization and processing of the 3D models were performed using the free Meshmixer software [28]. For a more realistic representation of the clinical situation of the patient, a soft tissue model created by an intraoral scan was superimposed on the 3D model created using the CBCT data.

Recently, new periodontal regeneration techniques have been proposed, the most important of which involve the generation of 3D printed scaffolds. A necessary condition is the highly accurate representation of the complex morphology of the periodontal defect of each patient, thus offering personalized treatment of the disease. For the successful generation of 3D periodontal scaffolds, it is necessary to adopt new combinatorial technological approaches, such as a combination of high-precision 3D printing with modern imaging techniques, such as CBCT. Studies conducted by the medical community on the diagnostic value of CBCT data for periodontal defects have shown their usefulness in detecting certain types of periodontal defects [29] and their superiority over digital X-rays for certain types of periodontal defects (e.g., grade I furcation involvements, three-wall defects, etc.) [30]. The first clinical case of periodontitis treatment in a patient using a 3D printed scaffold designed using CBCT data was performed in 2015 by Rasperini et al. [31]. The STL repair software Materialise Magics [32], the product design software Siemens NX [33], and the 3D medical image processing software Materialise Mimics [34] were used. Favorable results were obtained up to 12 months after the scaffold was placed on the patient. However, the research conducted on the 3D design of periodontitis scaffolds using CBCT data is very limited.

This article aims to fill the gap in the state-of-the-art research concerning the usage of CBCT data for 3D modeling of the hard tissues of the oral cavity of patients with periodontitis, for the need of designing and printing 3D scaffolds for periodontal regeneration. In this context, it aims to determine an optimal and efficient methodology for creating 3D models of teeth and alveolar bone, especially for being used as the basis for creating bioabsorbable 3D printed scaffolds of personalized treatment against periodontitis, which will simultaneously be used as sustained release drug carriers. Thus, in this article, segmentation and 3D modeling workflows using existing software packages are evaluated for the 3D reconstruction of the hard tissues of the oral cavity using CBCT data that belong to a patient with periodontitis, and comparisons between the 3D models generated through the experiments that yielded the most satisfactory results are made and the proposed 3D modeling workflow is discussed in detail, for being used for the needs of designing 3D scaffolds for periodontal regeneration.

## 2. Materials and Methods

### 2.1. Test Dataset and Evaluated Software

The data processed in this study were the anonymous DICOM (Digital Imaging and Communications in Medicine) data of the CBCT scan of a patient who has been diagnosed with periodontal disease by his/her dentist. The authors had no other previous knowledge of the patient or other relevant information on the diagnostic or treatment parameters. The entire patient’s maxilla was included in the imaging volume and was centered in the imaging volume. The CBCT parameters were reported to the authors as outlined in the following.

- CBCT scanner: NEWTOM VGI evo CBCT machine (CEFLA, Imola, BO, Italia).
- Imaging protocol: high resolution (HR) protocol with a voxel size of 150 microns (0.15 mm).
- Exposure settings: 110 KV and a total of 109.2 mAs.
- Field of view: standard 100 mm × 100 mm.
- Scanning procedure: based on manufacturer’s recommendations.

The 3D Slicer open-source medical image editing software, version 4.11.20210226 [26] was used in all experiments for image segmentation and generation of a first version of the 3D model of each segment of the oral system, defined by the segmentation. The Geomagic Wrap 2017 commercial software package (3D Systems, Rock Hill, SC, USA–now developed and supported by Artec3D, Luxemburg City, Luxemburg) [35] was used for processing the 3D reconstruction generated by 3D Slicer and generating the final 3D models.

### 2.2. Manual Segmentation

**Figure 1:**
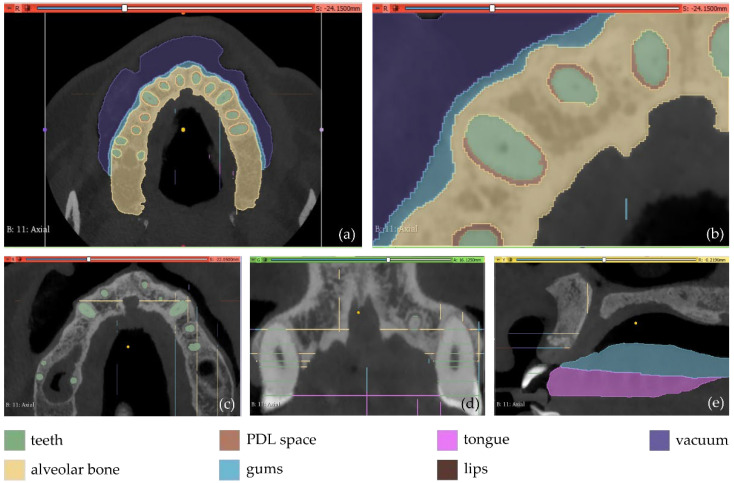
Manual definition of segments in 3D Slicer using CBCT slices of the maxilla of a patient within Experiment 1; (**a**): a CBCT slice in the axial plane; (**b**) zoom-in view of the CBCT slice in the axial plane, shown in (**a**); (**c**) zoom-in view of another CBCT slice in the axial plane; (**d**) zoom-in view of a CBCT slice in the coronal plane; (**e**) zoom-in view of a CBCT slice in the sagittal plane.

The first experiment (Experiment 1) involves the definition of 7 different segments, as described in the following (Figure 1).

1. Teeth: a segment for all teeth of the maxilla of the patient.
2. Alveolar bone: a segment for the alveolar bone of the maxilla of the patient.
3. Periodontal ligament space (PDL space), i.e., the soft tissue union between teeth and the alveolar bone: a segment for the PDL space of the maxilla of the patient.
4. Gums: a segment for the gums of the maxilla of the patient.
5. Tongue: a segment for the tongue of the patient.
6. Lips: a segment for the upper lips of the patient
7. Vacuum: a segment for the vacuum inside the maxilla of the patient, depicted through black areas in the CBCT scan.

**Figure 2:**
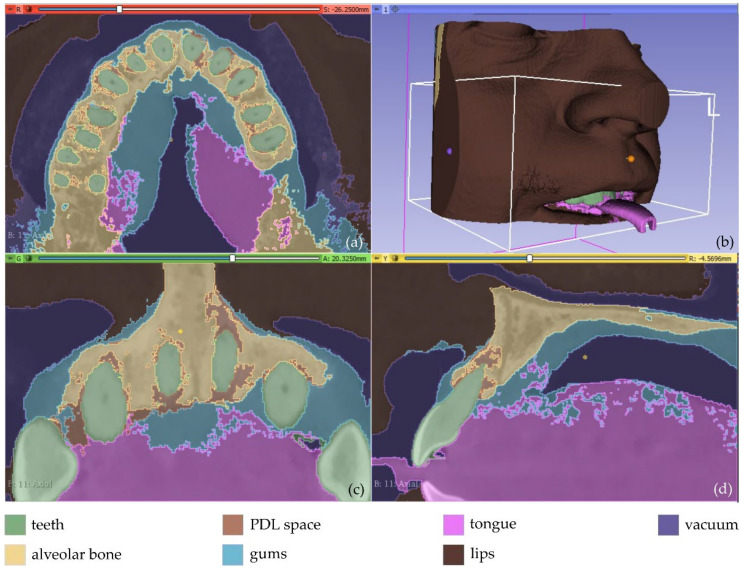
Segmentation results for Experiment 1 after the application of the “Grow from seeds” method via 3D Slicer, superimposed over CBCT slices in the axial plane (**a**), the coronal plane (**c**), and the sagittal plane (**d**), and 3D reconstructed volumes derived by the segmentation (**b**).

The aforementioned segments were defined in a few slices (<15 slices) by manual painting either parts of them or the entire segments where they were visible. The “Grow from seeds” method available in 3D Slicer was applied using the painted parts of segments in the defined slices. This method starts from these “seeds”, i.e., the painted segments inside each anatomical structure, and grows them to achieve a complete segmentation, using a variation of the GrowCut algorithm [36] designed for medical images, as described in [37]. The results of the “Grow from seeds” method in a few CBCT slices along with the 3D reconstructed volumes derived by the segmentation are shown in Figure 2.

**Figure 3:**
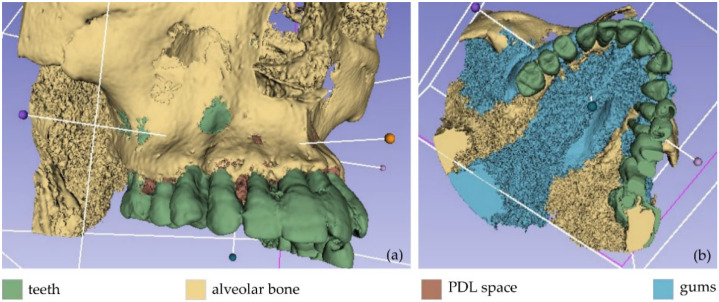
Non-processed 3D models as obtained by Experiment 1; (**a**): teeth, alveolar bone, and PDL space; (**b**) teeth, alveolar bone, PDL space, and gums.

The result of the segmentation is characterized by errors and a high percentage of noise, as illustrated in Figure 2. A 3D model for each segment was generated using the 3D Slicer software, as shown in Figure 3. The conclusions summarized in the following emerged from Experiment 1.

- The segments for the gums, the tongue, and the lips were not properly separated due to their similar intensity in the CBCT images.
- The segment for the PDL space was not correctly defined. This soft tissue union between the teeth and the alveolar bone occupies a very small percentage of the tomographic images (e.g., see Figure 1b). Also, in some images, it is visible while in others it is not visible. Thus, the automatic segmentation method could not yield satisfactory results for the PDL space.
- A big percentage of noise is present in the results, especially due to the segment defined for the PDL space.
- The manual accurate definition of the aforementioned segments took too much time.

**Figure 4:**
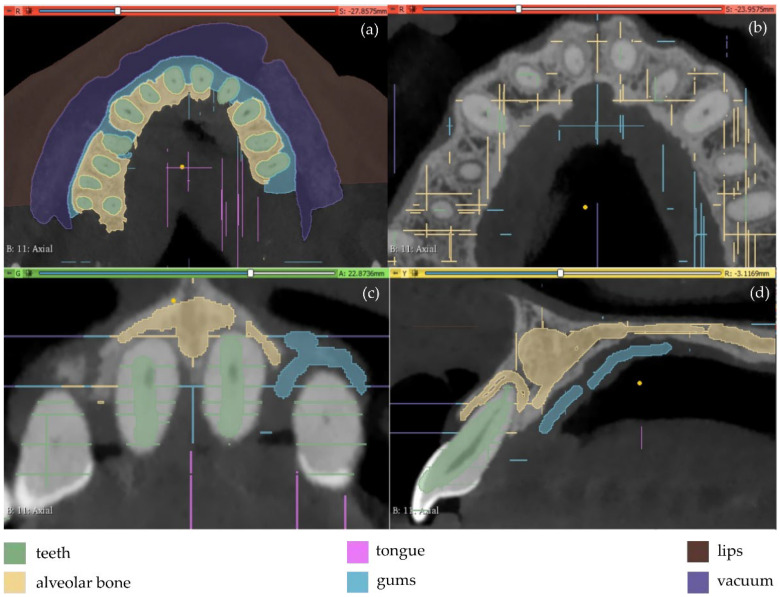
Manual definition of segments in CBCT slices (Experiment 2); (**a**,**b**): axial plane; (**c**): coronal plane; (**d**) sagittal plane.

In overall, the examined methodology was proved to be inefficient, without yielding accurate results for the needs of the present research. Thus, another experiment took place (Experiment 2). In the context of Experiment 2, the segments defined for the PDL space and the alveolar bone, within Experiment 1, were merged into one segment (alveolar bone). Also, more samples were manually defined in the CBCT images. The new samples (teeth, alveolar bone, gums, tongue, lips, vacuum) were defined in a faster way than in Experiment 1, as the emphasis was given on the definition of a greater number of samples for the segments rather than on the careful definition of their boundaries. Snapshots of the manual definition of samples for the six segments are shown in Figure 4.

**Figure 5:**
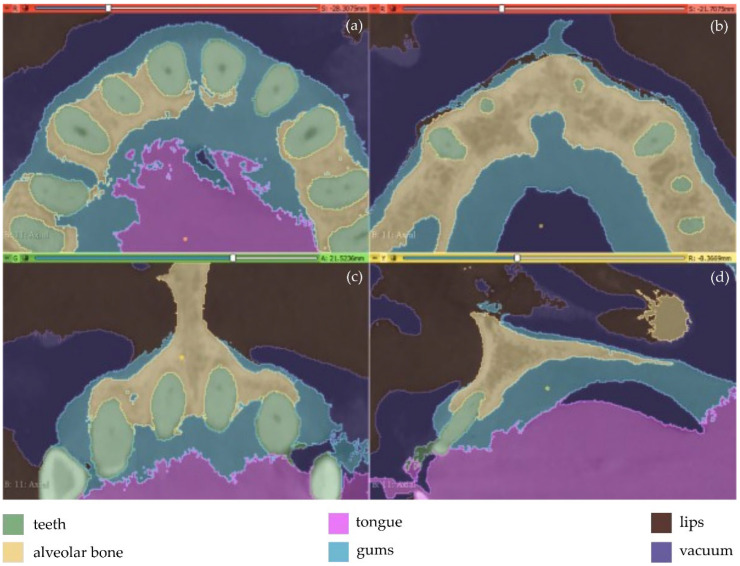
Segmentation results for Experiment 2 after the application of the “Grow from seeds” method, superimposed over CBCT slices in the axial plane (**a**,**b**), the coronal plane (**c**), and the sagittal plane (**d**).

The automatic “Grow from seeds” segmentation method available in the 3D Slicer software was then applied. The manually defined samples of the six segments were defined as input data to the method. After the initialization stage of the “Grow from seeds” method, an iterative process of correcting the results (through manually painting and erasing parts of segments) and updating them followed, until the segmentation results were deemed satisfactory. For instance, the segmentation results for four CBCT slices are shown in Figure 5. It is obvious that the segmentation produced much cleaner results in terms of noise than the segmentation applied in Experiment 1 (Figure 2).

**Figure 6:**
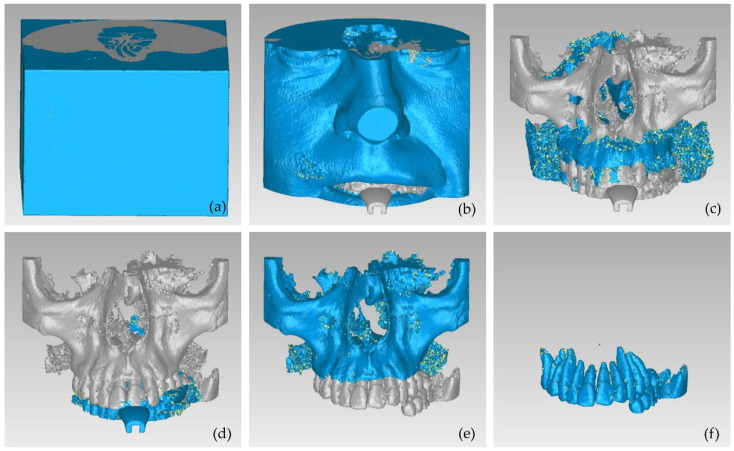
3D models resulting from the segmentation procedure conducted within Experiment 2, before being processed; (**a**): teeth, alveolar bone, gums, tongue, lips, vacuum (shown in blue); (**b**): teeth, alveolar bone, gums, tongue, lips (shown in blue); (**c**): teeth, alveolar bone, tongue, gums (shown in blue); (**d**): teeth, alveolar bone, tongue (shown in blue); (**e**): teeth, alveolar bone (shown in blue); (**f**): teeth.

Afterward, the results of the segmentation were converted into 3D meshes, i.e., a 3D model was produced for each segment, as shown in Figure 6. These 3D models need further processing, as they contain both outliers and noise. Thus, the 3D models of the teeth, the alveolar bone, the gums, and the lips were processed using the Geomagic Wrap software (the 3D model of the tongue was not processed further, as it was not required for the needs of the present study). The processing included the steps described below for each individual 3D model.

1. Conversion of the 3D surface model into a point cloud.
2. Processing of the point cloud, including, but not limited to, the stages described in the following.
  - Manual deletion of points outside the area of interest.
  - Manual and automatic removal of obviously wrong points (outliers).
  - Automatic noise removal.
  - Automatic reduction of the number of points in flat areas.
3. Generation of a 3D mesh model using the processed point cloud.
4. Processing of the 3D mesh model, including, but not limited to, the stages described in the following.
  - Automatic and manual closing of holes of the 3D surface.
  - Automatic noise reduction.
  - Automatic surface smoothing.
  - Automatic correction of non-manifold edges, self-intersections, and highly creased edges.
5. Storage of the 3D model in STL format.

**Figure 7:**
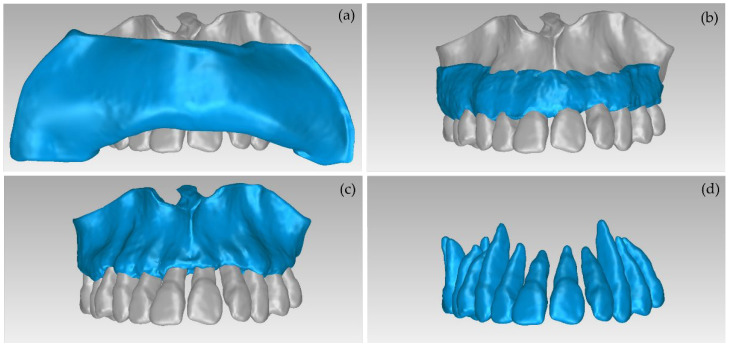
3D models of teeth, alveolar bone, gums, and lips after their processing via the Geomagic Wrap software; (**a**): teeth, alveolar bone, gums, lips (shown in blue); (**b**): teeth, alveolar bone, gums (shown in blue); (**c**): teeth, alveolar bone (shown in blue); (**d**): teeth.

These final 3D models, generated via the aforementioned procedure, are illustrated in Figure 7.

The conclusions obtained from Experiment 2 regarding the manual definition of segments followed by the automatic segmentation method “Grow from seeds” and a processing stage of the generated 3D models are outlined in the following. The process followed by Experiment 2 is quite time-consuming. Indicatively, the average time required to accurately mark the segment of teeth in a CBCT slice was 12 min. The average time required to accurately mark all segments in a CBCT slice was 30 min. The time required for the implementation of the entire segmentation process (i.e., the manual definition of segments and application of the “Grow from seeds” method with simultaneous correction of the segments after its initialization stage and updating of the results) was greater than 15 h. Therefore, this method was deemed inefficient for application to the entire maxilla or mandible. However, in the context of the present research, there is no need for 3D modeling of the entire maxilla or mandible, but only of the area suffering from periodontitis, for the generation of the 3D scaffold.

### 2.3. Automatic Segmentation

Due to the long time required for the manual definition of samples for each segment for use as input data in the “Grow from seeds” segmentation method, as observed in the two aforementioned experiments, an automatic segmentation experiment was carried out, as described in this section. Specifically, in the context of Experiment 3, an initial segmentation was performed automatically by applying a thresholding method for each segment using different thresholds. Four segments were defined, as mentioned below, along with the corresponding thresholds of the brightness values of the CBCT slices used:

- teeth: >1700.
- alveolar bone: [500, 1000].
- soft tissues: [−30, 300].
- vacuum: <−70.

**Figure 8:**
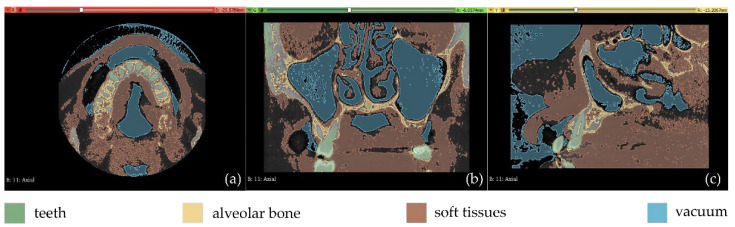
Automatic thresholding results (Experiment 3); (**a**): axial plane; (**b**): coronal plane; (**c**) sagittal plane.

**Figure 9:**
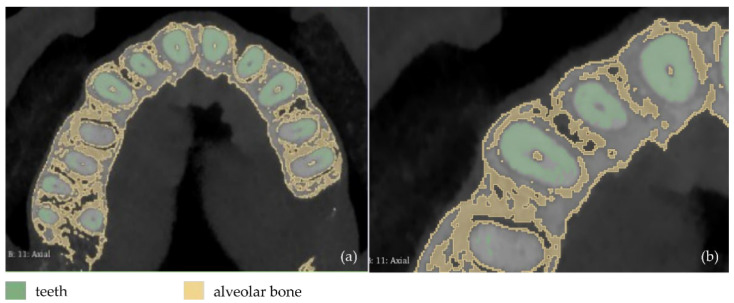
Automatic thresholding results for the segments defined for the teeth and the alveolar bone (Experiment 3); (**a**): a CBCT slice in the axial plane; (**b**): zoom-in view of (**a**).

**Figure 10:**
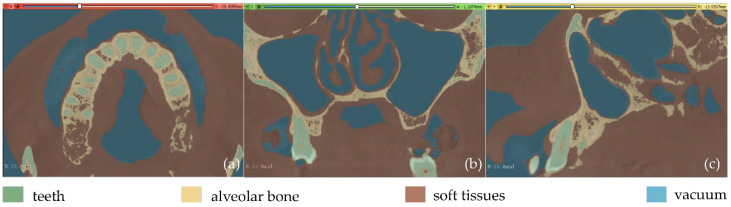
Segmentation results for Experiment 3 after the application of the “Grow from seeds” method, superimposed over CBCT slices in the axial plane (**a**), the coronal plane (**b**), and the sagittal plane (**c**).

**Figure 11:**
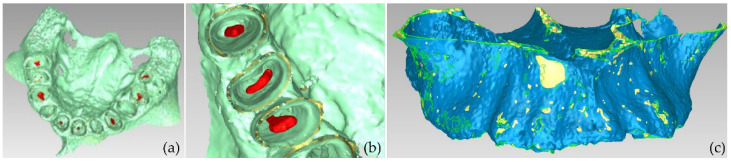
Views of the 3D model of the alveolar bone during its processing phase (Experiment 3).

**Figure 12:**
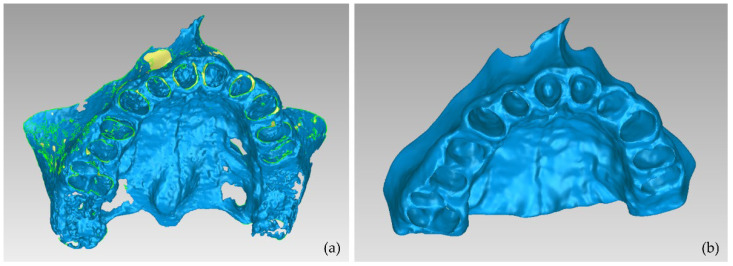
3D model of the alveolar bone; (**a**): the result of Experiment 3 (automatic segmentation); (**b**) result of Experiment 2 (manual segmentation).

Fewer segments were defined than those ones of the previous experiments, because the differentiation between gums/tongue/lips is not of interest in the context of the present research. What is more, a bigger number of segments means an increase in the 3D modeling time. Thus, a general segment, namely soft tissues, that includes the gums, tongue, and lips, was defined within Experiment 3. The results of the application of the automatic thresholding method for CBCT slices in the axial, coronal and sagittal planes are shown in Figure 8. Figure 9 shows the results of the thresholding method for the segments defined for the teeth and the alveolar bone, which are of particular interest in the context of the proposed research, as the 3D modeling of the hard tissues of the oral system is required. It is obvious that the application of a thresholding method solely does not yield good segmentation results; thus, another region growing technique has to be applied. Hence, the “Grow from seeds” method was applied using as input data the thresholding results. Figure 10 shows the results of the “Grow from seeds” segmentation method, which was carried out automatically without implementing any stage of processing and updating its results. The 3D model, for each of the generated segments, was very noisy. Figure 11 shows the 3D model of the alveolar bone during its processing phase within the Geomagic Wrap software environment, where some incorrect areas that need to be deleted are highlighted in red. As may be seen in Figure 11, the 3D model of the alveolar bone has many “problems”, including a large number of outliers, noise, self-intersections, holes, and non-manifold edges. Its processing lasted a long time without noticeable improvement in the result. Hence, Experiment 3, i.e., the methodology of automatic thresholding followed by an automatic “Grow from Seeds” method without any supervision and/or manual correction of the results, was abandoned. In Figure 12, the 3D models of the alveolar bone obtained manually and automatically through Experiments 2 and 3, respectively, are shown for visual comparison.

### 2.4. Semi-Automatic Segmentation

The manual segmentation workflow applied within Experiment 2 was inefficient in terms of time and human effort. In addition, the fully automatic segmentation workflow applied within Experiment 3 did not yield satisfactory results, as the obtained 3D models were not deemed to be satisfactory and needed a great amount of manual editing. Thus, a further experiment was performed (Experiment 4), where the segmentation was performed in a semi-automatic manner, combining automatic and manual methods available in the 3D Slicer software. Taking into account the fact that (a) in the context of this research, only the hard tissues of the oral system are of interest, and the 3D modeling of the soft tissues is not required (for the needs of the digital design of the scaffolds for periodontal regeneration) and (b) as it was concluded from the aforementioned experiments, the more segments are defined, the more manual processing they need, only three segments were defined within Experiment 4:

- teeth;
- alveolar bone;
- a segment including all the other regions of the oral cavity (except for the teeth and the alveolar bone), hereinafter referred to as “other”.

The procedure followed within Experiment 4 is described in the following.

- Automatic teeth thresholding (the threshold used for the brightness values of the CBCT slices: >1700).
- Automatic alveolar bone thresholding (brightness values of the CBCT slices range: 500–1000) and manual definition of more samples in areas where the alveolar bone was not recognized by thresholding.
- Manual definition of samples for the segment “other” (including gums, tongue, lips, vacuum, etc.).

**Figure 13:**
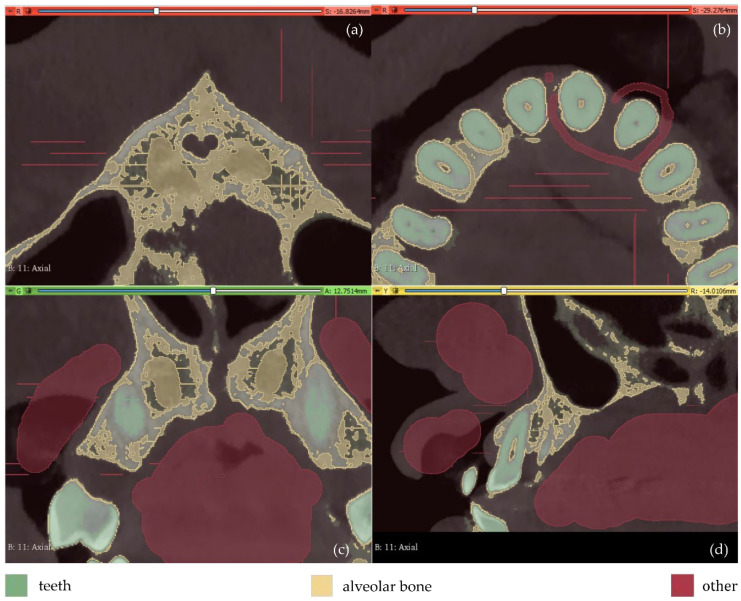
Manual definition of segments in CBCT slices (Experiment 4); (**a**,**b**): axial plane; (**c**): coronal plane; (**d**) sagittal plane.

**Figure 14:**
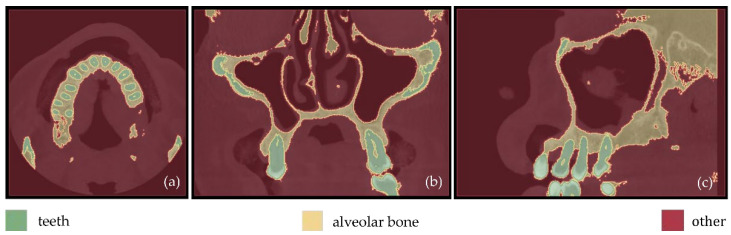
Segmentation results for Experiment 4 after the application of the “Grow from seeds” method, superimposed over CBCT slices in the axial plane (**a**), the coronal plane (**b**), and the sagittal plane (**c**).

The results are shown in Figure 13. From this image, it appears that a thin line around the teeth has been incorrectly identified as the alveolar bone. This is obvious in all three planes (axial, coronal, and sagittal planes), but it is more clearly visible in the axial plane (Figure 13b) where the thin line characterized as alveolar bone is present around all teeth. Using as input data the results of the aforementioned methodology, the “Grow from seeds” method was initialized and a process of repetitive manual processing of the segments and automatic updating of the segmentation results was applied, until the segmentation results were deemed to be satisfactory (Figure 14).

**Figure 15:**
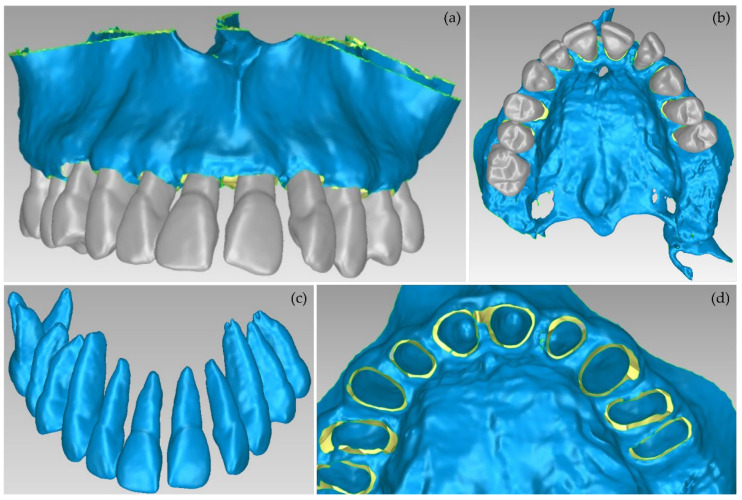
3D models of teeth and alveolar bone after their processing using the Geomagic Wrap software package (Experiment 4); (**a**,**b**): alveolar bone and teeth; (**c**): teeth; (**d**): alveolar bone.

The aforementioned segmentation methodology was followed by the extraction of the 3D models of the alveolar bone and the teeth through the 3D Slicer software and their processing in the Geomagic Wrap software, using the methodology mentioned in Section 2.2. The results are shown in Figure 15. The 3D models of teeth and alveolar bone generated via the workflow followed within Experiment 4 were deemed to be satisfactory. What is more, their generation, via the semi-automatic procedure of Experiment 4, required less time and effort, compared to Experiment 2 (manual segmentation).

## 3. Results

In order to obtain a quantification and visualization of the differences between the 3D models of teeth and alveolar bone that were generated through the aforementioned experiments that yielded satisfactory results, i.e., Experiment 2 (manual segmentation) and Experiment 4 (semi-automatic segmentation), some comparisons took place using the open-source software CloudCompare, version 2.11.2 [38]. These comparisons and their results are presented in this section.

### 3.1. 3D Models of Teeth

**Figure 16:**
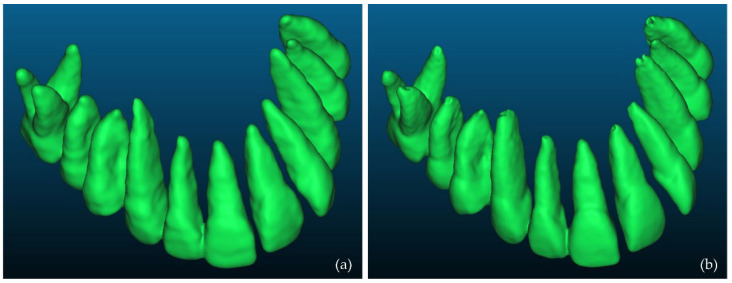
3D models of teeth after their processing using Geomagic Wrap; (**a**): Experiment 2 (manual segmentation); (**b**): Experiment 4 (semi-automatic segmentation).

**Figure 17:**
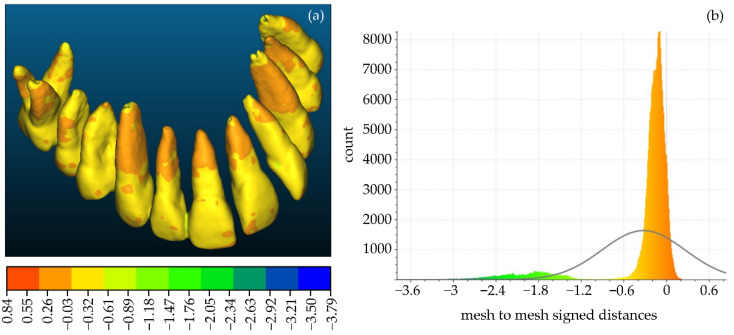
(**a**): Differences between the manually segmented 3D model of teeth of Experiment 2 (reference model) and the semi-automatically segmented 3D model of teeth of Experiment 4 (comparison model), visualized in the comparison model; (**b**) the histogram of the distances between the aforementioned 3D models of teeth.

Firstly, a comparison between the manually segmented (Experiment 2) and semi-automatically segmented (Experiment 4) 3D models of teeth was performed using CloudCompare. Specifically, the manually segmented 3D teeth model of Experiment 2, after being processed using the Geomagic Wrap software, was considered the reference 3D model. The semi-automatically segmented 3D teeth model of Experiment 4, similarly after being processed using the Geomagic Wrap software, was considered as the comparison model. These two 3D models are shown in Figure 16. Figure 17 shows the differences between these two 3D models, visualized in the comparison model (Experiment 4), as well as the histogram of the signed distances between the 3D models of teeth of Experiment 2 and Experiment 4. A total of 90% of the calculated distances (i.e., 239,807 out of 266,804) ranged between −0.7 mm and 0.2 mm, while the standard deviation of the distances was calculated to be 0.58 mm. These metrics were considered to be acceptable for the needs of the proposed research, which leads to the conclusion that a semi-automatic segmentation methodology such as the one adopted within Experiment 4 may be applied for 3D modeling of the hard tissues of the maxillofacial system with sufficient accuracy for the needs of creating scaffolds for periodontal regeneration.

### 3.2. 3D Models of Alveolar Bone

**Figure 18:**
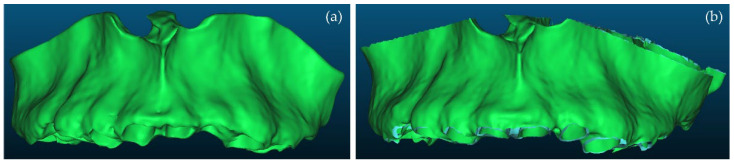
3D models of alveolar bone after their processing using Geomagic Wrap; (**a**): Experiment 2 (manual segmentation); (**b**): Experiment 4 (semi-automatic segmentation).

**Figure 19:**
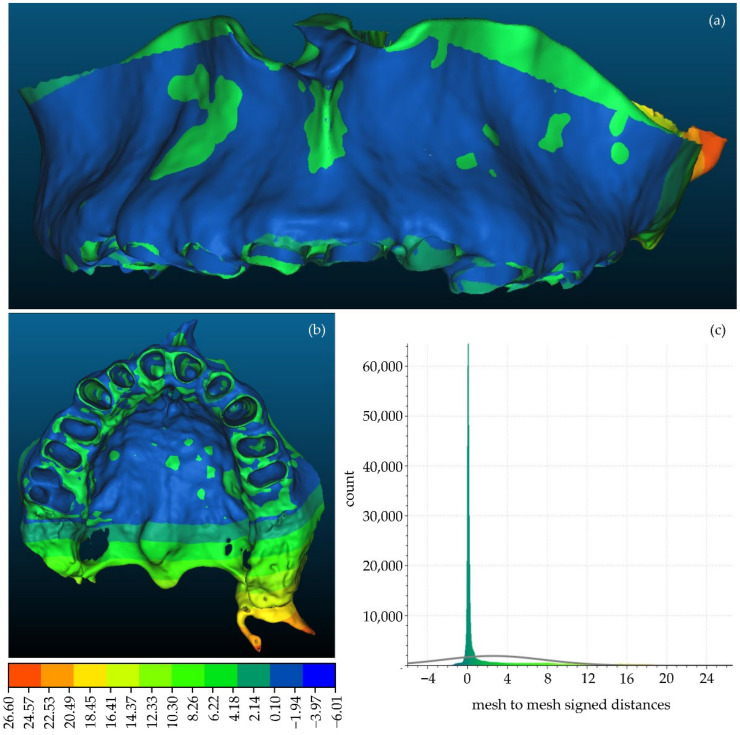
(**a**,**b**): Differences between the manually segmented 3D model of teeth of Experiment 2 (reference model) and the semi-automatically segmented 3D model of teeth of Experiment 4 (comparison model), visualized in the comparison model; (**c**) histogram of the distances between the aforementioned 3D models of teeth.

A comparison between the manually segmented (Experiment 2) and semi-automatically segmented (Experiment 4) 3D models of the alveolar bone was also performed, similarly using CloudCompare. The manually segmented 3D model of the alveolar model generated within Experiment 2, after being processed using the Geomagic Wrap software, was considered as the reference 3D model. The semi-automatically segmented 3D model of the alveolar bone, generated within Experiment 4, similarly after being processed using Geomagic Wrap, was considered as the comparison model. These two 3D models are shown in Figure 18. Figure 19 shows the differences between these 3D models, visualized in the comparison model (Experiment 4), as well as the histogram of the signed distances between the 3D models of the alveolar bone of Experiment 2 and Experiment 4. A total of 90% of the calculated distances (i.e., 502,670 out of 556,006) ranged between −2 mm and 11 mm, while the standard deviation of the distances was calculated to be 5.05 mm. These metrics, despite being worse than the corresponding metrics derived for the 3D models of teeth, were considered to be acceptable for the needs of the proposed research, mainly because the smallest differences are observed in the areas of interest, i.e., in areas with periodontal defects, while the biggest differences are observed in areas out of interest for the needs of the proposed research.

## 4. Discussion

The experiments conducted regarding the 3D modeling of the hard tissues of the oral cavity (and in some experiments of the soft tissues as well) which are presented in Section 2 of this article, and the comparisons of the 3D models of the hard tissues of the oral system (teeth and alveolar bone) that resulted from the tested manual and semi-automatic segmentation methodologies, which are presented in Section 3 of this article, contributed in the definition of the proposed 3D modeling methodology for the needs of the proposed research. In particular, from the experiments conducted, it was concluded that the fully automatic segmentation methodology (Experiment 3) cannot yield satisfactory results for the needs of 3D modeling of the hard tissues of the oral system. Also, the adopted fully manual segmentation (Experiment 2) was found to be very time-consuming and inefficient. Thus, the combination of automated and manual segmentation methods was proved to be the most efficient combination for the needs of the current research. The best combination of individual segments that should be defined for the needs of the proposed research are those ones used within the semi-automatic segmentation experiment (Experiment 4), i.e., (a) teeth; (b) alveolar bone; and (c) “other”, that is, a segment including all the other regions of the oral cavity (except for the teeth and the alveolar bone).

From the automatic and semi-automatic segmentation experiments (Experiment 3 and Experiment 4, respectively), it was found that a thresholding method produces automated results very quickly. However, the sole application of a thresholding method is not sufficient for the needs of segmentation. Moreover, it was proved that if thresholding is applied automatically to the whole set of CBCT images (i.e., if the same threshold is applied to the whole set of CBCT images), as it was conducted within Experiment 4, then some areas around the teeth are wrongly characterized as alveolar bone (e.g., Figure 13, thin line, wrongly labeled as the alveolar bone around the teeth). Despite the fact that these errors are corrected through the processing of the generated 3D models, further optimization of the adopted semi-automatic segmentation methodology was pursued. Thus, several experiments were conducted to optimize the use of the thresholding method using real CBCT datasets. It was concluded that the most efficient use of the thresholding result is its usage as a “mask” that guides the manual definition of samples of segments, based on the intensity values that belong to the used upper and lower thresholds. This methodology aims at overcoming the limitation of using the same threshold in all CBCT images. At the same time, the manual definition of samples is very fast, since the mask defined from the thresholds guides the definition of samples. What is more, greater precision is achieved in the boundaries of the manually defined samples of segments.

The procedure of thresholding individual CBCT images is sufficient to be applied to a small subset of the CBCT data (e.g., to approximately 30 CBCT images: ~10 in the axial plane, ~10 in the coronal plane, and ~10 in the sagittal plane). The automatic thresholding needs to be applied separately for the teeth and alveolar bone segments, while the segment that includes all the other regions of the oral cavity (“other”), can be roughly defined manually, in a similar number of CBCT images. The existence of a segment that includes the remaining regions of the oral cavity apart from the teeth and the alveolar bone is necessary for the application of the region growing segmentation method “Grow from seeds”, using as input data the samples of the three aforementioned segments. In case there was no segment including the remaining regions of the oral cavity apart from the teeth and the alveolar bone (i.e., segment “other”), then the segment for the alveolar bone, after the application of the “Grow from seeds” method, would occupy the entire area of each CBCT image, except for the area of the teeth. Thus, thanks to the definition of the segment “other”, the result of the “Grow from seeds” method is a complete segmentation of the CBCT dataset, with three segments: (a) a segment with distinct boundaries for the teeth; (b) a segment with distinct boundaries for the alveolar bone; and (c) a segment “other” with all the other regions of the oral system. Obviously, the “other” segment is no longer used for the needs of 3D modeling, as 3D models are only generated for the teeth and the alveolar bone segments.

After the initialization of the “Grow from seeds” method, it is necessary to correct its result through visual inspection, by deleting samples, inserting additional samples, or modifying the existing ones and updating the result of the method, until the visual inspection concludes that the result is satisfactory. In addition, it was found that the amount of manual work required increases with an increase in the area over which the segmentation process is applied. Therefore, before starting the segmentation process, it is very important to limit the segmentation area, by cropping the volume of interest in the CBCT dataset.

In accordance with the above, the proposed 3D modeling methodology of the hard tissues of the oral cavity using dental CBCT data for the needs of designing scaffolds for periodontal regeneration is outlined in the following.

1. Definition of the region of interest by cropping the corresponding volume defined in the CBCT dataset, using the 3D Slicer software. In this way, each CBCT image will depict only the region of interest, in all three reference planes (axial, coronal, sagittal).
2. Definition of the following three segments for the segmentation process within the 3D Slicer software:
  - teeth
  - alveolar bone
  - “other”, including all the other areas of the oral system, depicted in the CBCT images after their cropping, which do not belong to the teeth and alveolar bone segments.
3. Definition of samples for the three segments (teeth, alveolar bone, and other) for a small subset of CBCT images (e.g., 30 images: 10 in each reference plane). This procedure for each selected CBCT image may be performed as described below, using the 3D Slicer software.
  - Thresholding of the CBCT image using a data-dependent threshold, so that the teeth segment is defined. Indicatively, an average lower threshold for the intensity values of the CBCT image that may be used is ~1000, which is better determined through testing on the specific CBCT image.
  - Usage of the result of thresholding performed in step (a) as a mask and definition of the segment of teeth in the specific CBCT image using the “Paint” tool, only in areas constrained by the mask, i.e., in regions with intensity values defined by the threshold used in step (a) (e.g., >1000). Simultaneously, the “Erase” tool may optionally be used, to erase areas incorrectly labeled as teeth.
  - Thresholding of the CBCT image using a data-dependent threshold, so that the alveolar bone segment is defined. Indicatively, an average lower threshold for intensity values of the CBCT image that may be used is ~500, which is better determined through testing on the specific CBCT image.
  - Usage of the result of thresholding performed in step (c) as a mask and definition of the part of the alveolar bone in the specific CBCT image using the “Paint” tool, only in areas outside the teeth segment that are constrained by the mask, i.e., with intensity values defined by the threshold used in step (c) (e.g., >500). Hence, in this step, the teeth segment is also used as a mask that prevents the definition of the alveolar bone segment in the areas occupied by the teeth segment. Simultaneously, the “Erase” tool may optionally be used, to erase areas incorrectly labeled as the alveolar bone.
  - Manual (coarse) definition of the segment “other”, in the areas not occupied by the teeth and alveolar bone segments. It is recommended to use the teeth and alveolar bone segments as masks, so that they prevent the definition of the segment “other” in the areas they occupy. Simultaneously, the “Erase” tool may optionally be used, to erase areas incorrectly labeled as “other”.
4. Initialization of the “Grow from seeds” method available in the 3D Slicer software.
5. Visual inspection of the result of the “Grow from seeds” method and—if necessary—correction of the existing samples of the three segments (teeth, alveolar bone, and “other”) using the “Paint” and “Erase” tools of the 3D Slicer software.
6. Update of the result of the “Grow from seeds” method if at least a correction was made to at least one of the segments, in the 3D Slicer software.
7. Repetition of steps 5–6, until the visual inspection of the result of the “Grow from seeds” method does not show any “big” error, i.e., an error which is not easily corrected by editing the 3D model that will be generated using the segmentation output. Errors/noise that may be corrected more easily and/or in a faster way using the Geomagic Wrap software are recommended to be ignored.
8. Conversion of the segmentation results for the teeth and alveolar bone segments into 3D models (meshes) and storage of these models in STL format, using the 3D Slicer software.
9. Editing of the two 3D models (teeth and alveolar bone) using the Geomagic Wrap software. The processing of each 3D model may include, indicatively, the steps outlined in the following.
  - Conversion of the 3D mesh to point cloud.
  - Editing of the point cloud, which may include, but is not limited to, the steps outlined in the following.
    - Manual deletion of points outside the area of interest.
    - Manual and automatic removal of obviously incorrect points (outliers).
    - Automatic noise removal.
    - Automatic reduction of the number of points in flat areas.
  - Creation of a 3D mesh (surface) using the edited point cloud.
  - Editing of the 3D mesh, which may include, but is not limited to, the steps outlined in the following.
    - Automatic and manual closing of holes of the 3D model.
    - Automatic noise reduction.
    - Automatic surface smoothing.
    - Automatic correction of non-manifold edges, self-intersections, and highly creased edges.
10. Optional merging of the two 3D models (teeth and alveolar bone) into a single 3D model using the Geomagic Wrap software.
11. Storage of each processed 3D model (or the merged 3D model) in STL format, using Geomagic Wrap.

**Figure 20:**
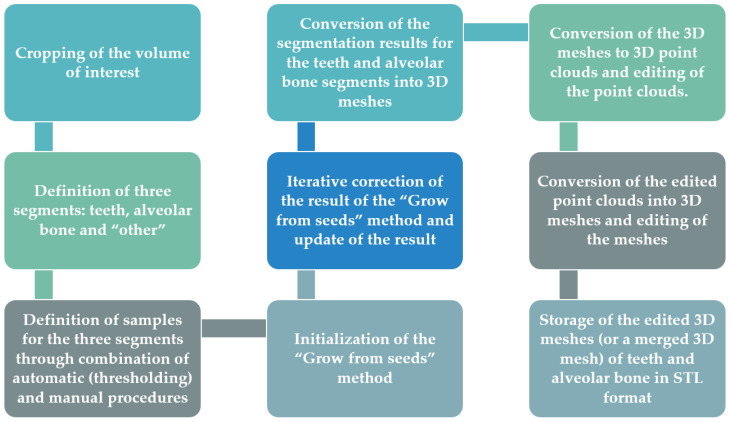
Proposed methodology for 3D modeling of the hard tissues of the oral system using dental CBCT data.

This process is summarized in Figure 20.

## 5. Conclusions

The purpose of this article was the determination a methodology for generating 3D models for the hard tissues of the oral cavity using dental CBCT data from patients with periodontitis, that will be used for designing and printing 3D scaffolds for periodontal regeneration. In order to obtain an optimized 3D modeling methodology for the needs of the proposed research, several experiments were conducted, which are described in detail in Section 2 of this article, while comparisons between the results of the experiments were also made, which are analyzed in Section 3 of this article. Finally, in Section 4, the proposed 3D modeling methodology is documented and described in detail. More specifically, in this article, the issues outlined in the following were addressed.

- Segmentation and 3D modeling workflows using existing software packages were evaluated for the 3D reconstruction of the hard tissues of the oral cavity using CBCT data that belong to a patient with periodontitis. Specifically, one medical image editing software solution was used for determining the optimal segmentation methodology that produces the best results in terms of efficiency and accuracy, and one 3D model editing software was used for generating the final 3D models. The main purpose of the evaluation conducted within this research was to determine the best combination of segmentation steps, including the optimal number of segments to be determined and the optimal combination of methods (automatic, semi-automatic, or manual ones) for generating 3D models that may be easily edited within a 3D editing software. The used software for segmentation and 3D model editing are only indicative; other software packages that include similar functions may also be used, following the proposed workflow.
- Comparisons between the 3D models generated through two—out of the four—experiments that yielded the most satisfactory results were made. These comparisons include the calculation of the distances between the two 3D models of the teeth and the two 3D models of the alveolar bone and their visualization (a) within a histogram and (b) in three dimensions as well, using one of the compared 3D models as a reference one. The comparisons aimed to obtain a rough estimation of the differences between these 3D models, so that it may be concluded if these differences are within permissible limits and if they may be considered negligible or insignificant for the needs of 3D modeling of the teeth and alveolar bone for periodontal regeneration. Indeed, the differences were considered to be acceptable for the needs of the proposed research, which leads to the conclusion that a semi-automatic segmentation methodology may be applied instead of a fully manual process. However, for a real comparison of the quality of the segmentation, a Micro CT dataset released for science and research would be useful to be used as a reference, thanks to the ultra-high resolution that it provides.
- The proposed 3D modeling workflow was discussed in detail, for being used for the needs of designing 3D scaffolds for periodontal regeneration. This workflow includes the definition of three segments for the segmentation process (teeth, alveolar bone, and “other”, including all the other areas of the oral system), the combination of automatic and manual stages for completing the segmentation process, and the usage of 3D editing tools for repairing the generated 3D model. Taking into account the fact that a CBCT dataset from only one patient was examined, the proposed 3D modeling workflow should be verified in a future research study using a greater number of CBCT datasets.

Future work will include the determination of a methodology for designing 3D scaffolds for being used in periodontal regenerative treatment, an investigation into new biomaterials for the generation of innovative bioabsorbable 3D printed scaffolds of personalized treatment against periodontitis, which will simultaneously be used as sustained release drug carriers, and the experimental 3D printing of such scaffolds for multiple differing cases of patients with periodontitis.

## Data Availability

Not applicable.
